# Supplementary material for: Routine and Advanced Laboratory Tests for Hemostasis Disorders in COVID-19 Patients: A Prospective Cohort Study
Source: J Clin Med. 2022 Mar 3;11(5):1383. doi: 10.3390/jcm11051383 (PMC8911406; doi:10.3390/jcm11051383)
Supplement: Supplementary file 1 [file jcm-11-01383-s001.zip › Table S1.pdf]

Supplementary Materials

**Table S1:** reference values for the laboratory tests	
**Parameters**	**Normal values**
Hemoglobin	>12g/dl
Reticulocytes	20-100 G/L
Platelets	150-400 G/L
Immature platelets	4.4-23.2 G/L
Leukocytes	4-10 G/L
Neutrophils	1.7-7.5 G/L
Lymphocytes	1.2-4 G/L
Monocytes	0.2-1 G/L
Fibrinogen	2-4 g/L
D-Dimers	<500 µg/L
vWF : GP1b-binding activity	50-250 UI/dL
Prothrombin Fragment 1+2	<290 pM
Prothrombin time ratio	75-100%
aPTT ratio	0.8-1.15
TGA	
Lagtime 5pM	2.5-3.75 min
ETP 5pM	1023-2104 nM.min
Peak 5pM	122-422 nM
TTP 5pM	4.8-8.7 min
Velocity 5pM	23.1-191.1 nM/min
Lagtime 20pM	1.7-2.4 min
ETP 20pM	1197-2395 nM.min
Peak 20pM	269-490 nM
TTP 20pM	3.8-5.3 min
Velocity 20pM	92.7-223.2 nM/min
ViscoElastic Testing	
CTH	109-150 s
CS	13-33.2 hPa
PCS	14.2-29.8 hPa
FCS	1.6-3.7 hPa

aPTT: activated prothrombin time; CS: clot stiffness; CTH: clotting time with heparinase; ETP: endogenous thrombin potential; FCS: fibrinogen contribution to CS; PCS: platelets' contribution to CS; TGA: thrombin generation assay; TTP: time to peak vWF: von Willebrand factor
