# Supplementary figures and images for: Routine and Advanced Laboratory Tests for Hemostasis Disorders in COVID-19 Patients: A Prospective Cohort Study
Source: J Clin Med. 2022 Mar 3;11(5):1383. doi: 10.3390/jcm11051383 (PMC8911406; doi:10.3390/jcm11051383)

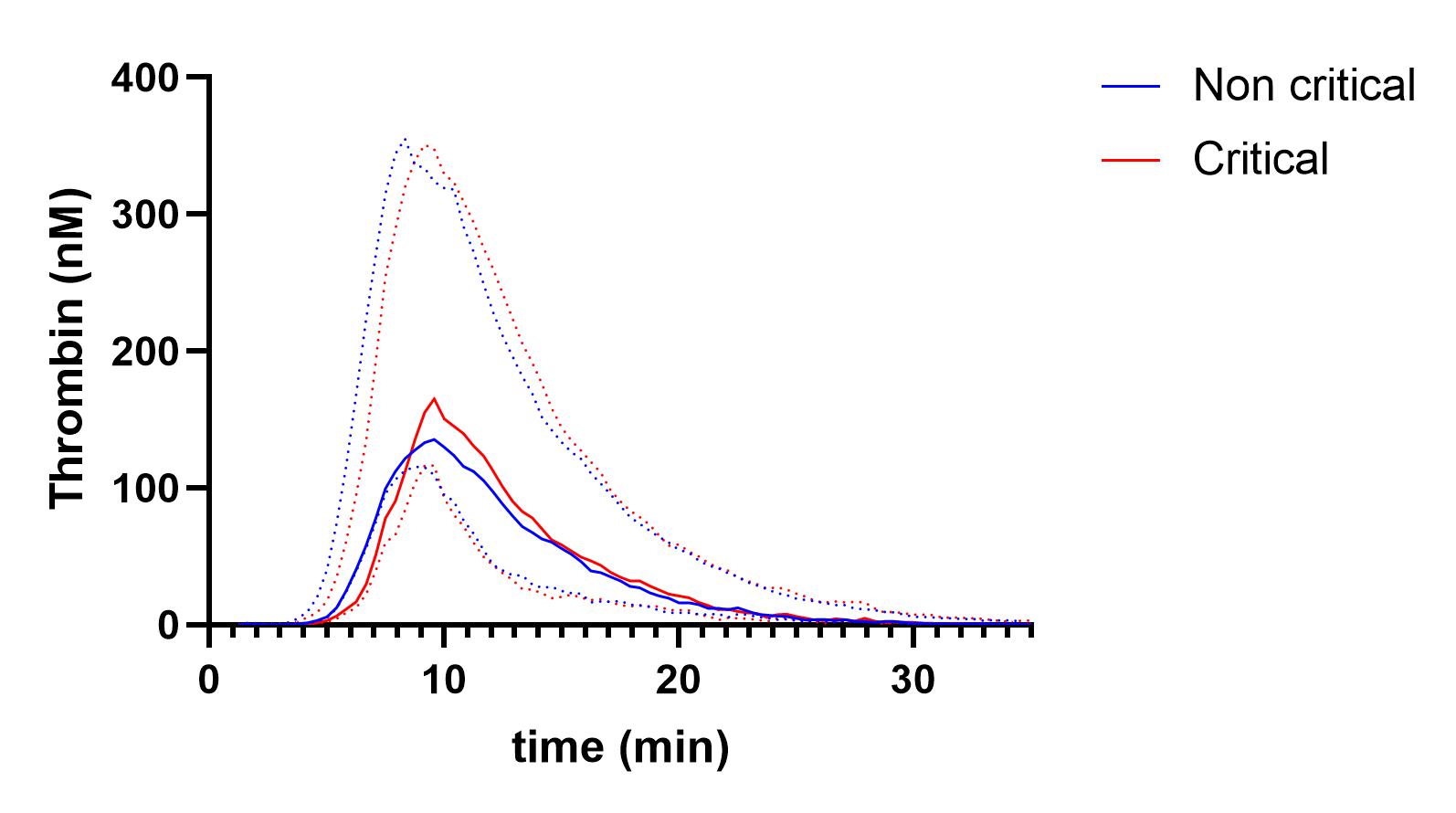

Supplement: Supplementary file 1 [file jcm-11-01383-s001.zip › Supplementary Figure S1.jpg]
